# Spectrophotometric Estimation of Ethamsylate and Mefenamic Acid from a Binary Mixture by Dual Wavelength and Simultaneous Equation Methods

**DOI:** 10.4103/0250-474X.40345

**Published:** 2008

**Authors:** Anju Goyal, I. Singhvi

**Affiliations:** Department of Pharmaceutical Chemistry, B. N. P. G. College of Pharmacy, Udaipur - 313 002, India; 1 Department of Pharmaceutical Sciences, M. L. Sukhadia University, Udaipur - 313 001, India

**Keywords:** Ethamsylate, mefenamic acid, simultaneous analysis, two wavelength calculation method, simultaneous equation method

## Abstract

Two simple, accurate, economical and reproducible spectrophotometric methods for simultaneous estimation of two-component drug mixture of ethamsylate and mefenamic acid in combined tablet dosage form have been developed. The first developed method involves formation and solving of simultaneous equation using 287.6 nm and 313.2 nm as two wavelengths. Second developed method is based on two wavelength calculation. Two wavelengths selected for estimation of ethamsylate were 274.4 nm and 301.2 nm while that for mefenamic acid were 304.8 nm and 320.4 nm. Both the developed methods obey Beer's law in the concentration ranges employed for the respective methods. The results of analysis were validated statistically and by recovery studies.

Ethamsylate (ESLT) is diethylammonium-2,5-dihydroxybenzenesulphonate and is used as a haemostatic agent for prevention and treatment of capillary hemorrhage associated with haemostasis, menorrhagia and post-partum haemorrhage^1^. The drug is official in British Pharmacopoeia with estimation of drug by potentiometric method^2^. Literature survey reveals that one spectrophotometric^3^ and one LC^4^ methods are reported for the estimation of ESLT from pharmaceutical formulations.

Mefenamic acid (MFNC) is 2-[(2,3-dimethylphenyl) amino]benzoic acid and is used as an analgesic and antiinflammatory agent^5^. The drug is official in British Pharmacopoeia with estimation of the drug by non-aqueous titrimetric method^6^. Literature survey reveals that one spectrophotometric^7^, one HPLC^8^ and three LC^9^–^11^ methods have been reported for the estimation of MFNC from pharmaceutical formulations. However none of the methods is yet reported for simultaneous estimation of two drugs from combined pharmaceutical dosage forms.

Developed spectrophotometric methods were found to be simple, rapid, accurate, reproducible and economical in comparison to routine extractive or colorimetric methods used for analysis of single drug and have been used successfully for determination of two components from combined tablet dosage form.

A PC based Systronic, UV/Vis double beam spectrophotometer (model No. 2101) with spectral bandwidth of 2 nm and wavelength accuracy ±0.5 nm (with automatic wavelength correction) and wavelength readability 0.1 nm increment was employed for all measurements using a matched pair of 10 mm quartz cells.

Standard bulk drug samples of ESLT and MFNC were provided by Ochoa Laboratories Pvt. Ltd., New Delhi. Methanol was used as solvent for the preparation of stock solution and for further dilutions. The tablet samples of combined dosage form of ESLT and MFNC [Sylate-M250 (Emcure Pharmaceuticals Ltd., Pune), Sylate-M500 (Emcure Pharmaceuticals Ltd., Pune) and Eklot-MF (Kontest Pharmaceuticals Ltd., Mumbai)] were procured from the local pharmacy.

In the first method, pure drug sample of ESLT and MFNC were dissolved separately in methanol so as to give several dilutions of standard in concentration range of 0-50 μg/ml of each drug. All dilutions were scanned in wavelength range of 200.0 450.0 nm. Two wavelengths selected for formation and solving of simultaneous equation were 287.6 nm and 313.2 nm. Absorptivity coefficient of both the drugs was determined at selected wavelengths. Absorptivity coefficient for ESLT at 287.6 nm and 313.2 nm were 37.40 cm^−1^ g^−1^ l and 157.78 cm^−1^ g ^−1^ l while respective values for MFNC were 328.80 cm^−1^ g^−1^ l and 111.62 cm^−1^ g^−1^ l. Set of two simultaneous equation thus framed were, A_1_ = 157.78 C_1_ + 111.62 C_2_-I and A_2_ = 37.40 C_1_ + 328.80 C_2_-II, where A_1_ and A_2_ are absorbance of sample solution at 287.6 nm and 313.2 nm, respectively. C_1_ and C_2_ are concentration of ESLT and MFNC, respectively in sample solution in g/l. Validity of above framed equation was checked by preparing five mixed standards using pure sample of two drugs, measuring their absorbance at respective wavelengths and calculating concentration of two components. The result of validation studies was found satisfactory.

Twenty tablets were accurately weighed and average weight per tablet was determined. Tablets were grounded to fine powder and tablet powder equivalent to 100 mg ESLT was weighed and extracted four times with 20 ml portions of methanol and filtered through Whatman filter paper no. 41 into a 100 ml volumetric flask. Washed residue with methanol and added washings to filtrate, volume of filtrate was made to 100 ml mark with methanol. From above filtrate 10 ml was diluted to 100 ml with methanol and finally 1 ml was further diluted to 10 ml with methanol. Absorbance of this final dilution was measured at 287.6 nm and 313.2 nm, respectively, and concentration of two drugs in the sample was calculated using above framed simultaneous equations-I and II. Results of analysis of tablet formulation are reported in Table 1.

**TABLE 1:** RESULTS OF ANALYSIS OF COMMERCIAL FORMULATION

Method	Batch	Label claim mg/Tab.		% Label claim estimated*		Standard deviation		% Recovery**	
					
		ESLT	MFNC	ESLT	MFNC	ESLT	MFNC	ESLT	MFNC
Method I	A	500	500	100.43	101.10	0.6066	0.2586	100.54	100.78
	B	250	250	100.74	100.96	0.3524	0.3601	101.10	100.83
	C	250	250	100.36	100.90	0.5788	0.1289	101.22	101.00
Method II	A	500	500	99.81	100.38	0.9200	0.9585	100.18	99.86
	B	250	250	100.73	101.34	0.9170	0.9600	100.40	101.63
	C	250	250	101.64	99.42	0.9150	0.9433	101.86	99.82

A is Sylate-M500 (Emcure Pharmaceuticals Ltd., Pune), B is Sylate-M250 (Emcure Pharmaceuticals Ltd., Pune) and C is Eklot-MF (Kontest Pharmaceuticals Ltd., Mumbai). ESLT is ethamsylate; MFNC is mefenamic acid.

* Each value is an average of five estimations;

** Average of recovery studies at three different concentration levels. Method I is simultaneous equation method; Method II is two wavelength calculation method

For method II, set of two wavelengths λ_1_ (274.4 nm) and λ_2_ (301.2 nm) for estimation of ESLT and λ_3_ (304.8 nm) and λ_4_ (320.4 nm) for estimation of MFNC were selected on basis of the principle that absorbance difference between two points on a mixture spectra is directly proportional to concentration of component of interest and independent of interfering component. Five mixed standards of pure drugs containing different concentration of ESLT and MFNC were prepared in methanol. All standards were scanned at respective set of selected wavelengths. Absorbance difference was measured and respective calibration curve was plotted.

Tablet samples were prepared in a similar manner as for method I. Final dilution was analyzed by scanning at respective set of wavelength and absorbance difference values were noted and concentration of ESLT and MFNC was calculated from the respective calibration curve. Results of analysis are reported in Table 1.

To study the accuracy, reproducibility and precision for both the developed methods recovery studies were carried out by the addition of standard drug solution to pre-analyzed tablet sample with proper dilutions at three different concentration levels with in the range of linearity for both the drugs. Results of recovery studies were found to be satisfactory and are reported in Table 1. The proposed methods for simultaneous estimation of ESLT and MFNC in combined tablet dosage form were found to be simple, accurate, rapid and economical. The values of recovery were close to 100% indicating reproducibility of the methods.

First developed method involving formation and solving of simultaneous equation based on accurate determination of absorptivity coefficient of two drugs at two selected wavelengths. Once the equation is framed then it is just required to measure the absorbance of sample solution at selected wavelengths and few calculations that can be manually done. Framed equations were validated using laboratory prepared mixed standards of two drugs which gave satisfactory results.

Second developed method for simultaneous analysis of ESLT and MFNC from combined dosage form make use of two wavelength calculation so as to remove interference between two components. Proper selection of two wavelengths for estimation of a component is critical.

The results of analysis of two drugs from tablet formulation using both the developed methods were found close to 100 percent for both ESLT and MFNC, standard deviation was satisfactorily low indicating accuracy and reproducibility of the methods. Recovery studies were satisfactory which shows that there is no interference of excipients. The developed methods were found to be simple, rapid, accurate and can be used for routine estimation of two drugs from tablet formulations.
